# Does selection for short sleep duration explain human vulnerability to Alzheimer’s disease?

**DOI:** 10.1093/emph/eow035

**Published:** 2017-01-24

**Authors:** Randolph M Nesse, Caleb E Finch, Charles L Nunn

**Affiliations:** 1 School of Life Sciences and Center for Evolution and Medicine, Arizona State University, Tempe, AZ 85287, USA; 2 Leonard Davis School of Gerontology, University of Southern California, Los Angeles, CA 90089, USA; 3 Department of Evolutionary Anthropology, Duke Global Health Institute, Duke University, Durham, NC 27708, USA

**Keywords:** evolution, Alzheimer's disease, sleep, glymphatic system, amyloid beta, melatonin

## Abstract

Compared with other primates, humans sleep less and have a much higher prevalence of Alzheimer ’s disease (AD) pathology. This article reviews evidence relevant to the hypothesis that natural selection for shorter sleep time in humans has compromised the efficacy of physiological mechanisms that protect against AD during sleep. In particular, the glymphatic system drains interstitial fluid from the brain, removing extra-cellular amyloid beta (eAβ) twice as fast during sleep. In addition, melatonin—a peptide hormone that increases markedly during sleep—is an effective antioxidant that inhibits the polymerization of soluble eAβ into insoluble amyloid fibrils that are associated with AD. Sleep deprivation increases plaque formation and AD, which itself disrupts sleep, potentially creating a positive feedback cycle. These and other physiological benefits of sleep may be compromised by short sleep durations. Our hypothesis highlights possible long-term side effects of medications that reduce sleep, and may lead to potential new strategies for preventing and treating AD.

## INTRODUCTION

Why are older humans distinctive among the apes in their high prevalence of Alzheimer’s disease (AD) [1]? The prevalence of AD increases exponentially in humans, from under 2% at age 60 to about 40% of individuals over age 90 [2]. Although its deleterious effects typically manifest too late in the life span to have a major influence on Darwinian fitness (reproductive success), the high prevalence of this severely debilitating and often fatal neurodegenerative brain disease in old humans, and wide variations in the vulnerability of different species, motivate us to seek an evolutionary explanation [3–5].

In particular, among apes, humans are distinctively vulnerable to the neuronal damage associated with AD [1,6], as initially hypothesized by Stanley Rapoport [7]. While older great apes acquire modest levels of amyloid beta (Aβ) deposits at ages younger than they are observed in humans, these amyloids are typically diffuse and not associated with degenerating neurons. In contrast, neuritic changes and AD are commonly associated with plaques in humans [1,8]. Humans are also outliers in their dramatically shorter sleep time compared to other primates, as discussed below. These two facts intersect with new findings on the protective role of sleep to suggest that selection for short sleep duration may contribute to the distinctive human vulnerability to AD.

AD is characterized by progressive cognitive deficits, especially of short-term memory, that are associated with the loss of synapses and the death of specific groups of neurons. The pathological hallmarks of AD are gross shrinkage of the cerebral cortex and the presence of fibrillar amyloid in neuritic plaques, which are aggregates of Aβ. The Aβ peptide is produced throughout life by neurons and is normally present in brain in interstitial and cerebrospinal fluid, as well as in the peripheral blood [9]. AD is also characterized by neurofibrillary tangles of hyperphosphorylated tau protein within neurons.

The accumulation of brain amyloid fibrils and tau pathology can be detected by PET imaging before clinical grade dementia [10,11]. Neurofibrillary degeneration typically spreads during clinical AD from the medial temporal cortex into other cortical regions, and subcortically into the hippocampus, a key site of spatial memory; the earliest phase may emanate from the brain stem locus coeruleus [12,13]. While MRI comparisons of humans with great apes reveal a relatively larger frontal lobe in humans [14,15], neuroanatomical differences do not readily explain the severe neurodegenerative loss in humans during AD. Humans and other primates differ in patterns of gene expression in brain pathways subject to neurodegeneration [8,16], yet the amyloid peptide sequence is widely shared across vertebrates, and is identical in humans and primates [17].

Humans are also unique in their multiple isoforms of apolipoprotein E (ApoE2,-3, and -4), which differ in affinity for receptors and lipids, whereas other primates have a single isoform, E4 [18–20]. ApoE4 is the major risk factor for AD, while ApoE2 is AD-protective. Beside its role in blood cholesterol management, ApoE is important to the homeostasis and remodeling of brain synapses. ApoE4, which is considered the ancestral allele [20], shows selective advantage in resistance to infections [15].

Far from purely pathogenic, the amyloid precursor protein (APP) is cleaved into several peptides with diverse actions; some are neurotrophic, while others are neurotoxic during development and throughout life [21]. A broader perspective is emerging on the highly evolved functions of amyloid that go beyond the initial Aβ neurodegenerative cascade hypothesis in AD [22–24]. In particular, the 2010 suggestion that Aβ has anti-microbial activity [25] has recently been confirmed in studies showing that the expression of Aβ_40_ or Aβ_42_ in cultured cells extends survival in the presence of *C. albicans*, and that the expression of Aβ_42 in_ in transgenic *C. elegans* extends survival in the face of gut infection with *C. albicans* or *S. thyphimurium* [16]. Furthermore, the presence of microbes induces Aβ precipitation in the mouse brain within days, and Aβ forms fibrils that entangle fungi in a manner similar to other antimicrobial peptides [16]. Antiviral actions of Aβ have also been suggested, in conjunction with evidence that viral infections may contribute to AD [26].

## HUMANS SLEEP LESS THAN OTHER PRIMATES

Humans are also distinctive in their tendency to sleep considerably less than other primates. In this context, estimates of typical human sleep are critical: is the 8 h that sleep that physicians recommend also a good estimate for ancestral average human sleep duration? To address this question, researchers are studying traditional human populations that lack access to electricity, and thus likely have a stronger circadian drive based on natural cycles of light and darkness. One recent study found the average sleep times in three different hunter-gatherer groups range from 5.7 to 7.1 h, with an overall average of 6.5 h [27]. Another set of authors observed a nearly identical average sleep time for agriculturalists in rural Madagascar [28], while a study of a Haitian population lacking access to electricity identified the sleep duration as 7 h [29].

Notably, these studies used actigraphy, a method that is known to overestimate sleep durations [30]. In addition, napping is less common than expected in hunter-gatherers [27], yet perhaps more common and longer in agriculturalists, potentially adding up to an hour of sleep per day [28]. Taken together, 7 h is a good upper-level estimate of typical human sleep, with many ancestral populations likely sleeping less than this when ecological or social conditions constrain options for safe sleep. A meta-analysis including 65 studies of 3577 subjects in Western societies also found a 7.0 h average total sleep time [31].

**Figure 1.**
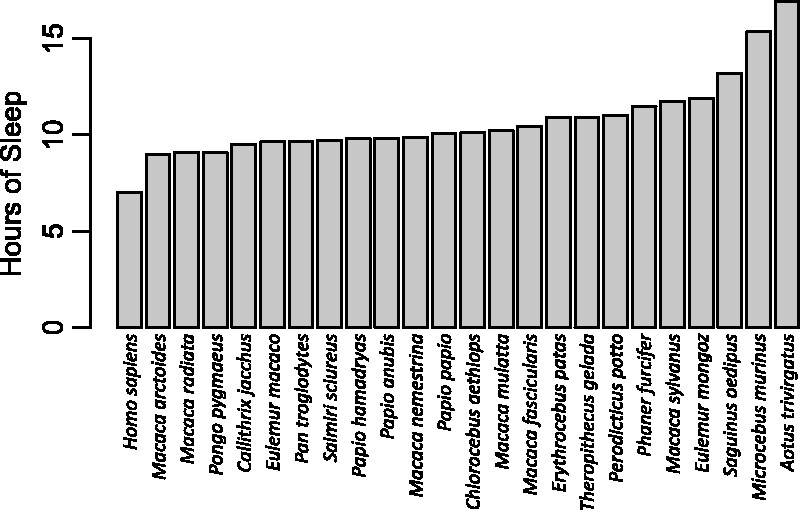
Duration of total sleep time in primates, including humans. Humans are the shortest sleeping primate (here, using a value of 7 h, see text). The data on nonhuman primates come from studies in which sleep was staged, most often by EEG, in adult animals, and thus exclude some studies based on videography [37] or studies of juvenile animals [38]

Using 7 h as a conservative estimate of the ancestral human sleep duration, humans are clearly short sleepers relative to other primates (Fig. 1). Yet, is this different from what one would expect for a diurnal primate with the body and brain size of a human? Samson and Nunn [32] investigated human sleep more quantitatively using a method that predicts a phenotypic characteristic of a particular species based on trait co-variation across the clade of interest, phenotypic characteristics of the ‘target’ species, and the phylogenetic placement of the species of interest [33–35]. The implementation of the method used by these authors is Bayesian, thus producing a posterior probability distribution of predicted sleep duration in humans. With this posterior distribution, one can compare the observed duration of sleep—7 h in this case—to the posterior distribution of predictions. If humans lie below the 95% credible interval of values in the distribution, they are determined to be a negative evolutionary outlier (or a positive outlier if above the 95% credible interval).

Using body mass, activity period, endocranial volume, percentage of leaves in the diet, interbirth interval and foraging group size as predictor variables, and a posterior distribution of primate evolutionary trees from a Bayesian phylogenetic analysis [36], Samson and Nunn’s [32] analysis predicted that that humans should sleep for an astonishing average of 10.3 h per night, with a 95% credible interval of 7.9–13.3 h per night. The conservative estimate of 7 h of sleep per night falls well below the 95% credible interval, suggesting that the average human sleeps much less than predicted for a primate with our phenotypic characteristics and the characteristics of our close evolutionary relatives.

Several selective pressures involving the risks and opportunity costs of sleep may have favored shorter sleep in humans [32]. In terms of risks, one selection pressure likely involved the transition from sleeping in the trees to sleeping on the ground, where risk of predation increased. While there is uncertainty about the rates of predator attacks on current hunter gatherers, predation rates for our ancestors were likely high [39]. In addition to increased vulnerability to predators, terrestrial sleep also makes humans more vulnerable to hostile conspecifics, both within the group and from other groups, because movement and attacks may be easier at night when on the ground.

Sleeping also imposes opportunity costs including lost chances to socialize, to learn from others or to learn through direct trial and error. Individuals who sleep less could engage in more social learning and social grooming, thus enhancing learning and formation of alliances. Individuals who sleep less could engage in more social learning and social grooming, thus enhancing learning and formation of alliances [40]. Interestingly, Samson and Nunn [32] also found that the percentage of rapid eye movement sleep (REM) was also higher than predicted for humans, which may enhance memory consolidation, mental rehearsal of social and environmental challenges in dreams, and general problem-solving within the shorter period of human sleep. Many of these phenomena would be beneficial for learning and social alliance formation, and for rehearsal of risks associated with terrestrial sleep.

## LINKS BETWEEN SHORT SLEEP, EVOLUTION AND AD

If selection for short sleep duration helps to explain increased human vulnerability to AD, then sleep disruption should speed AD onset and progression, mediated by definable and specific mechanisms. Recent research has confirmed the physiologic necessity of sleep, documented the dire health consequences of interrupted sleep and discovered several mechanisms that may mediate this relationship. We discuss each of these in turn.

### The utility of sleep

Sleep deprivation has well documented deleterious effects on health [41]. The need for sleep is a cross-species universal: extensive evidence confirms its necessity despite the costliness of sleep in terms of reduced vigilance [42]. Rats forced to stay awake are more vulnerable to bacterial infections and tumor growth [43]. It is broadly assumed that sleep facilitates repair of tissue wear-and-tear from daily activity because of severe health consequences of sleep-deprivation. Humans with fatal familial insomnia and other ‘circadian disruptions’ have higher mortality from diverse morbidities that impact mental and physiological functions [44,45].

### Sleep and AD

The role of sleep disruption in AD pathogenesis is difficult to assess because AD itself is associated with sleep disruption, as analyzed in humans and mouse models by David Holtzman and colleagues [46]. With this caveat in mind, self-reported sleep disturbance at age 70 in a large prospective sample of men showed 3-fold higher subsequent risk of AD, whereas sleep disturbance at age 50 did not influence risk [47]. A large prospective study found increased risk of compromised cognitive function for individuals who had previously reported fewer than 6 or more than 8 h of average sleep. However, the study also found worse health for those at the extremes, so the causal direction is unclear [48]. Moreover, in a very recent prospective study of a large sample of cognitively normal subjects followed for development of subsequent AD (National Alzheimer Coordinating Center), those with sleep disturbances had 3.4-fold higher risk of subsequent AD; this risk ratio was further increased by including BMI and APOE genotype as covariates, suggesting that sleep is an independent risk factor for AD [49]. Both reports are consistent with data showing greater Aβ accumulations measured via PET scan in those with shorter sleep in a community sample [50].

Sleep deprivation of mice also slightly increased Aβ in brain interstitial fluid [51]. Cross-sectional and longitudinal studies suggest that sleep disruption can be a cause and an effect of AD [46,52]. Holtzman’s group is generating further data which suggests a positive feedback cycle of sleep disruption that could accelerate neurodegeneration during AD [46,53]. On the positive side, sleep induction decreased axonal injury in a rat model of traumatic brain injury [54], and sleep may more generally reduce AD-related neuropathology [55].

## THE GLYMPHATIC SYSTEM

Recent research identifies some of the mechanisms underlying the utility of sleep and its potential connection to AD. The brain has a specialized lymphatic system involving astroglial cells, the ‘glymphatic system’, that channels interstitial fluids in the brain through astrocytes into the peripheral lymphatic system [56–59]. The glymphatic system is especially relevant to AD because it transports Aβ and metabolites out of the brain. The connection to sleep is also strong, as the Aβ transport rate is doubled during sleep [59]. This has led some scientists to hypothesize that insufficient sleep may be a vital factor in the progression of AD [57–60]. Disruption of glymphatic transport by inadequate sleep might also mediate other effects on AD. For instance, traumatic brain injury (TBI) often causes severe sleep disruption that may contribute to the premature development of amyloid plaques and tangles [61,62].

Aging may also impair glymphatic transport, as suggested by the 40% decreased clearance of brain Aβ in older mice [63]. Differences between species also provide useful evidence for a role for the glymphatic system removing metabolic products. A comparative study of cortical neuronal density in 24 mammalian species found an inverse correlation of sleep duration with the extra-cellular diffusion space. This finding was interpreted as an outcome of selection for the capacity to clear metabolites while maximizing the number of hours awake for foraging [64]. In related work, Barton and Capellini suggest that human sleep may have become more efficient because the risks of sleeping on the ground make it so costly [65].

## MELATONIN

MEL may also influence rates of AD progression. Levels of this potent antioxidant hormone increase more than ten-fold in darkness and in association with sleep onset. Reduced MEL levels, because of lack of sleep or sleeping with lights on, increases the risk of breast cancer [66], as confirmed in mice [67]. Levels are reduced in the CSF of patients with AD, and levels decrease further with disease progression [68]. The anti-inflammatory actions of melatonin are consistent with extensive evidence for the possible role of inflammation in the pathogenesis of AD. MEL has not only anti-inflammatory functions, but can also directly inhibit the *in vitro* fibrillization of Aβ [69,70]. By NMR spectrometry, MEL binds to Aβ at a single low affinity binding site, allowing the joint clearance of MEL-Aβ complexes [71]. In mice, melatonin protected against the neurotoxicity of Aβ [72], whereas administered MEL had several effects that decrease Aβ production [73]. *In vitro*, Aβ directly inhibits MEL production in mouse pineal cells *in vitro*.

## CONCLUSIONS

Collectively, these emerging findings document a role for sleep disruption in the pathogenesis of AD that supports our suggestion that selection for short sleeping time in humans may have made humans more susceptible to AD. We do not propose that the emergence of AD in human evolution has influenced Darwinian fitness. Fitness impacts are possible after the end of reproduction via kin selection, but they are unlikely in this case because AD is rarely manifested until after age 60, when selective forces on individuals are weaker. We consider AD to be an epiphenomenon of selection acting on other traits, focusing here on the trade-off between the special benefits for humans of reduced sleep time, and the associated costs, including decreased protection of the brain from AD and other kinds of damage.

While the data and recent findings generally point toward a link between the evolution of short sleep and AD in humans, we also wish to point out several caveats and limitations. First, the effects of variations in sleep for individuals *within* a species do not prove the effects of variations in sleep duration *across* species. In the several million years since the divergence of humans from our last common ancestor with other primates, selection may have adapted the sleep system to function more efficiently [65].

Second, while the proposed short-term social and safety benefits from shorter sleeping times are plausible, they have not yet been documented in humans. Data are also not yet available about possible social or physical disadvantages experienced by hunter gathers who sleep less than others, topics ripe for more research.

Third, the short sleep duration in humans could have other explanations; for example, changes in brain physiology associated with selection on cognitive function could increase sleep efficiency, resulting in less need for sleep. We must also consider why Aβ accumulations (but not dying neurons) appear years earlier in the brains of other primates compared to humans [1,17], who sleep much less.

Fourth, the brain differences that make humans more vulnerable to neuron damage are not direct results of Aβ accumulation. Neuronal damage instead seems to require interactions with immune mechanisms [74], which also mediate neuron pruning in the normal course of development [75]. Of special interest are aspects of innate immune function, particularly CD33, a receptor that regulates Aβ uptake by microglia and has uniquely evolved alleles in humans [76]. Strong selection on these systems in the course of rapid brain evolution offers an alternative explanation for the distinctive human vulnerability to AD [3].

Another important unanswered question is whether glymphatic function, and the more general physiological protection provided by sleep, vary considerably among individuals, and whether such variations are correlated with perceived need for sleep. If organisms have mechanisms that monitor the concentrations of brain metabolic products and modulate sleep need accordingly, then those who thrive on fewer hours of sleep are doubly fortunate. However, if maintenance of brain integrity is proportional to the absolute number of hours of sleep, healthy people who need less sleep may be more vulnerable to AD. We are unable to find reliable data on this point, but nonhuman animal studies of the effects of sleep time variations are possible. Also needed are studies of how glymphatic function, plaque formation and AD progression are influenced by new drugs that reduce the need for sleep.
